# Human cytomegalovirus interleukin-10 enhances matrigel invasion of MDA-MB-231 breast cancer cells

**DOI:** 10.1186/s12935-017-0399-5

**Published:** 2017-02-13

**Authors:** Cendy A. Valle Oseguera, Juliet V. Spencer

**Affiliations:** Department of Biology, University of San Francisco, 2130 Fulton Street, San Francisco, CA 94117 USA

**Keywords:** Breast cancer, Cytokine, Cytomegalovirus, cmvIL-10, Invasion

## Abstract

**Background:**

While some risk factors for breast cancer are well-known, the influence of other factors, particularly virus infection, remains unclear. Human cytomegalovirus (HCMV) is widespread in the general population, and both molecular and epidemiological evidence has indicated links between HCMV and breast cancer. The HCMV protein cmvIL-10 is a potent suppressor of immune function that has also been shown to promote proliferation and migration of breast cancer cells. In this study, the impact of cmvIL-10 on tumor cell invasion through a simulated basement membrane was investigated.

**Results:**

MDA-MB-231 breast cancer cells exhibited invasion through a matrigel layer that was significantly enhanced in the presence of either purified cmvIL-10 or supernatants from HCMV-infected cells containing secreted cmvIL-10. Transcriptional profiling revealed that cmvIL-10 altered expression of several genes implicated in metastasis. Exposure to cmvIL-10 resulted in higher *MMP*-*3* mRNA levels, greater protein expression, and increased enzymatic activity. Treatment with cmvIL-10 also increased expression of both urokinase plasminogen receptor (uPAR) and plasminogen activator inhibitor-1 (PAI-1), which can stimulate MMP-3 activity and have previously been identified as poor prognostic markers in breast cancer patients. Finally, MDA-MB-231 cells treated with cmvIL-10 showed significant downregulation of metastasis suppressor 1 (*MTSS1*), a scaffolding protein that regulates cytoskeletal rearrangements and is frequently lost in metastatic tumors.

**Conclusions:**

HCMV, and in particular the secreted viral cytokine, cmvIL-10, can induce cellular changes that facilitate cell migration and invasion. These findings indicate that HCMV may be associated with promoting the malignant spread of breast cancer cells and suggest that antiviral treatment may be a useful complement to chemotherapy in some patients.

**Electronic supplementary material:**

The online version of this article (doi:10.1186/s12935-017-0399-5) contains supplementary material, which is available to authorized users.

## Background

Breast cancer is the second leading cause of cancer deaths for women in the United States and a significant cause of mortality worldwide. While there are many known risk factors for breast cancer, infectious disease has emerged as one likely contributor to carcinogenesis [1, 2]. Recent studies have implicated a number of different viral infections in breast cancer, including bovine leukemia virus [3, 4], human mammary tumor virus [5], human papillomavirus [6], Epstein–Barr virus (EBV) [7–9], and human cytomegalovirus (HCMV) [10, 11]. Although there is no clear causal role for any of these viruses, a combination of molecular and epidemiological evidence suggests an association between HCMV and breast cancer.

HCMV is a β-herpesvirus that infects 70–90% of the general population, causing acute, persistent, or lifelong latent infection [12]. HCMV infections are typically subclinical and serious disease occurs mainly in immune-compromised individuals [12]. Overall, HCMV serostatus has not been positively correlated with breast cancer; however, women with breast cancer were found to have higher mean HCMV IgG levels in an Australian case–control study [13], suggesting that they might have experienced a recent infection. Analysis of a Norwegian cohort by the same group also revealed that elevation of HCMV IgG, but not EBV IgG levels, preceded the development of breast cancer in some women [14].

While serological evidence for HCMV in breast cancer may be limited, a stronger case is made by studies that have detected viral DNA and proteins by PCR and immunohistochemistry (IHC) in tumor biopsy specimens. In Taiwan, analysis of 62 breast cancer patients found that detection of both HHV-8 and HCMV in tumor samples by PCR was associated with lower overall survival and a decrease in relapse-free time [6]. Harkins et al. detected HCMV immediate early (IE) proteins by IHC in breast glandular epithelial cells in 31 of 32 specimens from patients with ductal carcinoma in situ (DCIS) or infiltrating ductal carcinoma (IDC) [10]. Another study found both HCMV IE and late proteins expressed in metastatic tumor cells in 100% of breast cancer specimens analyzed (73 total), and viral DNA was detected in 12/12 samples tested [11]. Detection of virus in breast epithelial cells is consistent with the notion that epithelial cells are a site of HCMV persistence [15], and further supported by the fact that transmission of infectious virus through breast milk is well-documented [16–19].

Despite the evidence indicating the presence of viral proteins and DNA in breast tumor tissue, HCMV is not typically considered an oncogenic virus [20]. Virus infection can, however, promote many of the classic hallmarks of cancer [21, 22], such as cell cycle dysregulation, inhibition of apoptosis, increased migration and invasion, and immune evasion [20, 23]. HCMV has been linked not only to breast cancer, but to an array of other malignancies, including glioblastoma [24–27], medulloblastoma [28], colon cancer [29], and prostate cancer [30]. Individual HCMV gene products can have profound effects on cell growth, such as immediate early proteins IE1 and IE2, which are known to stimulate entry into S phase [31, 32]. IE1 expression was found to increase the growth rate of glioblastoma cells in culture, suppress p53 and Rb tumor suppressor activity, and stimulate PI3K/Akt signaling [33]. IE1 was detected in breast tumor tissue [10, 11] as well as in CD133^+^ glioma stem cells isolated from glioblastoma multiforme (GBM) patients [34], suggesting that IE1 may promote tumorigenesis enhancing the growth and self-renewal of tumor stem cells.

Another HCMV gene implicated in tumor development is US28, which encodes a functional chemokine receptor that binds several human chemokines, including CCL2/MCP-1, CCL5/Rantes, and CX3CL1/Fractalkine [14, 35]. US28 also exhibits constitutive signaling activity, and cells expressing US28 are highly invasive [27] and form tumors in nude mice [36, 37]. US28 was found to induce vascular endothelial growth factor (VEGF), cyclooxygenase-2 (COX2), and Stat3 activation through upregulation of IL-6 [36–38]. Analysis of glioblastoma tumor specimens revealed the presence of both US28 and phosphorylated Stat3 [27, 38], demonstrating that US28 may play role in tumor development in vivo.

Whereas the US28 and IE1 gene products are expressed in infected cells, the UL111A gene encodes cmvIL-10, a viral cytokine that is secreted from infected cells. Although cmvIL-10 has only 27% sequence identity to human interleukin-10 (hIL-10) [39], the viral cytokine binds to the cellular IL-10 receptor with greater affinity than hIL-10 itself [40]. Extensive immunosuppressive properties of cmvIL-10 have been documented, including inhibition of inflammatory cytokine synthesis, downregulation of class I and II MHC, and inhibition of dendritic cell maturation [41–48]. Engagement of the IL-10 receptor by cmvIL-10 leads to activation of Stat3 [49–53], which is commonly constitutively activated in breast cancer cells [54], associated with poor prognosis in ovarian cancer, and considered a key factor in metastasis formation [55]. CmvIL-10 was found to activate Stat3 and play a pivotal role in the progression of malignant glioma by enhancing the invasiveness and migration of glioma cancer stem cells [56]. Because cmvIL-10 is secreted from the infected cell, it has the potential to act on any cell type, infected or not, that expresses the IL-10 receptor.

We have previously shown that the breast cancer cell lines MDA-MB-231 and MCF-7 express the IL-10R and that exposure to cmvIL-10 results in enhanced cell proliferation and migration [57, 58]. In the present study, we examined the impact of cmvIL-10 on MDA-MB-231 cell invasion through a simulated basement membrane and investigated the effect of cmvIL-10 on a panel of metastasis-related genes. We found that cmvIL-10 was a potent enhancer of invasion and influenced expression of genes strongly linked to the metastatic spread of breast cancer.

## Methods

### Cell, viruses, and reagents

MDA-MB-231 human breast adenocarcinoma cells (American Type Culture Collection, Manassas, VA) were cultured in L-15 Leibovitz’s Medium (Mediatech, Manassas, VA) supplemented with 10% fetal bovine serum (FBS) (Atlanta Biologicals, Flowery Branch, GA) at 37 °C with atmospheric CO_2_. The human foreskin fibroblast (HFF) cell line (ATCC) was cultured in Dulbecco’s Modified Eagle Medium (DMEM) with 10% FBS at 37 °C in a humidified chamber with 5% CO_2_. Human cytomegalovirus strain AD169 (ATCC) was used to infect confluent monolayers of HFFs at the indicated multiplicities of infection. Purified recombinant cmvIL-10, human IL-10, and epidermal growth factor (EGF) were purchased from R&D Systems (Minneapolis, MN). IL-10R neutralizing antibody and S3I-201 Stat3 inhibitor were from Santa Cruz Biotechnology (Santa Cruz, CA).

### Migration and invasion assays

Transwell migration was monitored using 96-well BD Fluoroblock plates with 8 µm filters (Corning, Inc., Corning, NY). Cells were harvested and suspended at density of 2 × 10^6^ cells/ml in migration media (L-15 + 1% FBS), and a volume of 75 μl cell suspension was placed on top of the filter inserts. Where indicated, IL-10R neutralizing antibody was added at a concentration of 30 μg/ml. The bottom wells were loaded with the indicated concentrations of EGF in the presence of conditioned medium from mock or HCMV-infected fibroblasts (96 h post infection) in a total volume of 235 μl. After 5 h at 37 °C, cells that traversed the filter and entered the lower chamber were quantified by the addition of Cell Titer Glo (Promega, Madison, WI) using a Turner Veritas luminometer. For invasion, 96-well matrigel-coated BD Fluoroblock transwell invasion plates (Corning) were used. Invasion plates were re-hydrated with warm media at 37 °C for 3 h and then 75 μl cell suspension loaded onto the hydrated filters as described above. Where indicated, 10 μM Stat3 inhibitor was included with cells in the top chamber; cmvIL-10, hIL-10 or conditioned medium was present in both chambers. The bottom plates received the indicated EGF concentrations, and then transwell system was incubated for 22 h at 37 °C with atmospheric CO_2_. At harvest, cells that had degraded the matrigel and entered the lower chamber were quantified by the addition of Cell Titer Glo as above.

### Quantitative PCR arrays

RNA was harvested from 10 × 10^6^ MDA-MB-231 cells that were mock treated or treated with 100 ng/ml cmvIL-10 or hIL-10 for 5 h using the RNeasy Midi Kit and RNAse-Free DNase set (Qiagen, Valencia, CA). From the isolated RNA, cDNA was prepared using the RT^2^ First Strand Kit (SA Biosciences, Frederick, MD) and subsequently loaded into a 96-well breast cancer metastasis profiler PCR array (PAHS-028ZD) with system RT^2^ SYBR Green Mastermix (SA Biosciences). The plates were run using the CFX96 Real-Time system cycler (BioRad, Hercules, CA) with the following amplification program: 95 °C for 10 min, 95 °C for 15 min with a slow ramp rate for 1.0 c/s and 60 °C for 1 min. The data from three biological replicates for each treatment was analyzed by the ΔΔCT method according to manufacturer’s instructions using the RT^2^ profiler PCR array data analysis program located on the SABiosciences web portal and is reported as fold change relative to control.

### Enzyme-linked immunosorbent assay (ELISA)

DuoSet ELISA kits (R&D Systems) were used to quantify uPAR, PAI-1, and MMP-3. For uPAR and PAI-1 measurement, MDA cells were seeded in triplicate in 96-well plate at 5.0 × 10^4^ cell/ml density with complete L-15 media and treated with 10 ng/ml of either cmvIL-10 or hIL-10 for the indicated times and supernatants were collected daily. The ELISA was carried out on supernatants according to manufacturer’s instructions using and following the addition of substrate and stop solution, absorbance of the plate was measured at 450 nm using a Dynex Opsys MR microplate reader. Sample concentrations were interpolated from a standard curve using linear regression analysis. For cell-associated MMP-3, MDA cells were seeded in 96-well plates and treated with cmvIL-10 as above. Cells were treated with cell lysis buffer (150 mM NaCl, 20 mM HEPES, 0.5% Triton-X-100, 1.0 mM NaOV_4_, 1.0 mM EDTA, 0.1% NaN_3_) supplemented with 1× protease inhibitors (Calbiochem, EMD Chemicals, San Diego CA) and were collected daily for the indicated time points. The lysates were evaluated for MMP-3 according to the manufacturer’s instructions (R&D Systems).

### Western blotting and zymography

Confluent T-75 flasks of MDA-MB-231 cells were treated with 10 ng/ml cmvIL-10 (R&D systems) for the indicated times, then scraped and harvested into cell lysis buffer (150 mM NaCl, 20 mM HEPES, 0.5% Triton-X-100, 1.0 mM NaOV_4_, 1.0 mM EDTA, 0.1% NaN_3_) containing 1× protease inhibitors (Calbiochem). Cell lysates were clarified via centrifugation, heated at 70 °C for 10 min in reducing buffer, and the proteins separated on a 4–12% Tris-Base SDS-PAGE gel (Life Technologies, Grand Island, NY). After transfer to nitrocellulose, the membrane was incubated in blocking solution (5% milk + TBS) for 1 h at room and then probed with primary antibody: 1:1000 dilution for MMP-3 or MTSS-1 antibodies (Santa Cruz), or MAPK antiserum (Cell Signaling Tech, Danvers, MA), in blocking solution overnight, oscillating on a platform rocker at 4.0 °C. After three washes, the membranes were incubated with a 1:2000 dilution of appropriate AP-conjugated secondary antibody on a platform rocker at room temperature for 1 h. Protein bands were detected using western blue stabilized AP substrate (Promega, Madison, WI). For zymography, cell lysates were denatured in SDS buffer under non-reducing conditions without heat, and run on a 4–16% Zymogram gel using Tris–Glycine SDS running buffer according to manufacturer’s instructions. After electrophoresis, the enzyme was renatured by incubating the gel in Zymogram Renaturing Buffer containing a non-ionic detergent, then equilibrated in Zymogram Developing Buffer (to add divalent metal cations required for enzymatic activity), and then stained and destained to reveal digested (clear) areas corresponding to active enzyme.

### Immunofluorescence microscopy

MDA-MB-231 cells were seeded onto FBS-coated glass coverslips at a density of 2.0 × 10^5^ cells/well and cultured for 48 h at 37 °C. Cells were treated with 100 ng/ml of purified recombinant cmvIL-10 for 96 h, then fixed with 2% paraformaldehyde in DPBS for 20 min, washed, permeabilized with 0.2% Triton-X-100 in PBS for 15 min. The cells were washed and blocked with 10% FBS for 1 h at 37 °C, then incubated with anti-MTSS-1 antibody at a 1:100 dilution for 1 h at 37 °C followed by the addition of goat anti-mouse TRITC secondary antibody at a 1:150 dilution for 30 min (Life Technologies) and Alexa Fluor 488 phalloidin (Molecular Probes, Eugene, OR). Coverslips were washed and excess fluid was removed before inverting the coverslip onto a glass slide containing 20 μl of DAPI-containing Prolong Gold mounting medium (Life Technologies, Grand Island, NY). Images were taken on a Zeiss AX10 Imager.A1 microscope (Carl Zeiss Inc., Oberkochen, Germany) using AxioVision 4.7.2 imaging software.

### Statistical analysis

Statistical analyses was performed using the paired, two-tailed Student’s t test.

## Results and discussion

**Fig. 1:**
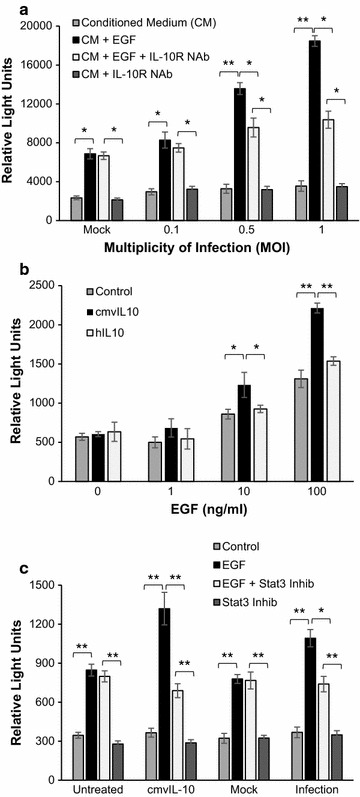
MDA-MB-231 cells exhibit increased migration and matrigel invasion in the presence of cmvIL-10. **a** Transwell migration toward EGF after 5 h in the presence of conditioned medium from mock or HCMV-infected fibroblasts at the indicated MOIs. IL-10R neutralizing antibody (NAb) was included at 30 μg/ml. **b** Matrigel invasion toward EGF in the presence of 100 ng/ml purified recombinant cmvIL-10 or hIL-10 after 22 h. **c** Matrigel invasion toward EGF in the presence or absence of 100 ng/ml purified recombinant cmvIL-10 or conditioned medium from mock or infected fibroblasts (MOI = 1). Where indicated, 10 μM Stat3 inhibitor was included. *Error bars* SEM. *p < 0.05, **p < 0.001. These results are representative of three independent experiments

The tumor microenvironment is a complex milieu that includes not only malignant cells, but immune cells, fibroblasts, signaling molecules, the extracellular matrix (ECM), and blood vessels. We have previously found that cmvIL-10 enhances migration of MDA-MB-231 breast cancer cells in vitro toward epidermal growth factor (EGF) [57]. In order to more faithfully replicate conditions under which cmvIL-10 might be found in the tumor microenvironment, we examined the ability of cmvIL-10 secreted from virus-infected cells to stimulate movement of MDA cells. Monolayer cultures of human foreskin fibroblasts were mock-infected or infected with HCMV strain AD169 at a range of multiplicities of infection (MOI). After 96 h, supernatants were harvested and placed in the lower chamber of a transwell migration plate in the presence or absence of EGF. MDA cells were placed in the upper chamber, separated from the EGF and conditioned medium by a porous filter. After five hours, cells that traversed the filter were quantified. MDA cells did not exhibit any significant movement toward conditioned medium from mock or infected cells, which is consistent with our previous finding that cmvIL-10 is not a chemoattractant for tumor cells [57]. However, when conditioned medium from mock infected cells was supplemented with EGF, cell migration was observed (Fig. 1a). When EGF was added to conditioned medium from HCMV-infected cells, the amount of cell migration increased, suggesting that substances released from virus-infected cells amplified chemotaxis to EGF. Moreover, the enhanced MDA cell movement was greater when EGF was provided in supernatants from higher MOI infections, and thus greater concentrations of cmvIL-10, indicating a dose-dependent effect. To confirm that cmvIL-10 was the virally produced substance mediating this increase in cell movement, MDA cells were pre-incubated for 30 min with a neutralizing antibody (NAb) directed at the cellular IL-10R. The NAb was also included in the top chamber with MDA cells during the 5 h incubation, and resulting migration was reduced to levels seen when only EGF was present in medium from mock infected cells. These results demonstrate that cmvIL-10 secreted from virally infected cells has the ability to interact with the cellular IL-10R on tumor cells to enhance directed movement.

To further recapitulate the tumor microenvironment, we examined whether cmvIL-10 could also promote invasion through matrigel, a gelatinous protein mixture derived from mouse sarcoma cells widely used to simulate the ECM in vitro [59]. MDA cells were place atop a matrigel-coated transwell system with EGF placed in the lower chambers. Purified recombinant cmvIL-10 or hIL-10 was added to both chambers. After incubation for 22 h, invasion was assessed by counting cells in the lower chamber, which should contain only the cells that were able to degrade the matrigel coating to access the porous filter. As shown in Fig. 1b, cmvIL-10 was found to be a strong enhancer of cell invasion. Surprisingly, cmvIL-10 was able to increase invasion of MDA breast cancer cells to a significantly greater extent than hIL-10, suggesting that the viral cytokine may trigger signaling events that are distinct from the cellular cytokine. Since activation of the transcription factor Stat3 by cmvIL-10 is well-documented [39, 49–51, 53, 60, 61], we next examined the need for Stat3 in cmvIL-10-enhanced invasion. Treatment with a Stat3 inhibitor reduced the cmvIL-10-induced increase in invasion through matrigel toward EGF seen when either recombinant purified protein or cytokine produced during virus infection were present (Fig. 1c). Taken together, these results demonstrate the novel finding that not only does cmvIL-10 produced during virus infection stimulate enhanced migration and invasion of breast cancer cells, but it does so more effectively than hIL-10.

**Fig. 2:**
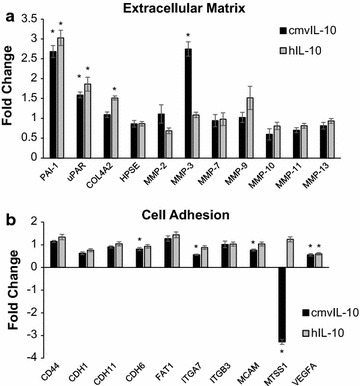
CmvIL-10 induces changes in metastasis-related gene expression in MDA-MB-231 cells. RNA was extracted from cells treated with 100 ng/ml cmvIL-10 or hIL-10 for 5 h, then RNA was purified and expression of 84 genes was analyzed using the Human Tumor Metastasis RT2 Profiler PCR Array. **a** Select genes encoding extracellular matrix proteins or **b** cell adhesion proteins is shown. Fold changes represent comparison to untreated MDA-MB-231 and are the average of three biological replicates. *Error bars* SEM. *p < 0.05. A complete list of genes analyzed is found in Additional file 1: Table S1

Given the impact of cmvIL-10 on MDA cell invasion, we wanted to investigate whether the viral cytokine brought about changes in the expression of genes associated with tumor metastasis. Transcriptional profiling was performed using a tumor metastasis qPCR array designed to analyze 84 genes known to be involved in breast cancer metastasis. MDA cells were mock-treated or incubated with either cmvIL-10 or hIL-10 for 5 h, then RNA was extracted, cDNA synthesized, and qPCR performed. Additional file 1: Table S1 contains a complete list of genes analyzed with fold changes for cmvIL-10 or hIL-10 treated cells compared to mock treated control cells indicated. Select genes encoding proteins associated with either the ECM (Fig. 2a) or cell adhesion (Fig. 2b) are shown graphically. Overall, plasminogen activator inhibitor (*PAI*-*1*) was the most highly upregulated gene for both cytokines, with expression increased by 2.68-fold by cmvIL-10 and 3.12-fold by hIL-10. Interestingly, increased expression of urokinase plasminogen receptor (*uPAR*) was also common to both cmvIL-10 and hIL-10 (1.59- and 1.87-fold increases, respectively). Matrix metalloproteinase-3 (*MMP3*) was specifically upregulated by cmvIL-10 only (2.75-fold increase), while collagen type 4 (*COL4A*) expression was increased by hIL-10 only (1.51-fold increase). Changes in cell adhesion genes were more modest, with only one gene, metastasis suppressor 1 (*MTSS1*) exhibiting a statistically significant change of more than twofold, and this was observed for cmvIL-10 treatment only (0.305-fold change, or −3.28). Slight decreases in integrin alpha 7 (*ITGA7*, 0.561- or −1.78-fold change), melanoma cell adhesion factor (*MCAM*, 0.768- or −1.32-fold change), and cadherin 6 (*CDH6*, 0.811- or −1.23-fold change) were also found with cmvIL-10 treatment. Both cmvIL-10 and hIL-10 induced a slight decrease in expression of vascular endothelial growth factor (*VEGFA*, 0.554- or −1.80-fold for cmvIL-10; 0.5987- or 1.67-fold change for hIL-10). Chemokine receptor CXCR2 expression was also strongly decreased by cmvIL-10 and hIL-10, but those changes were not statistically significant. Overall, these transcriptional profiling results indicate that cmvIL-10, as well as human IL-10, can affect expression of genes that are likely to promote metastatic spread of tumor cells.

**Fig. 3:**
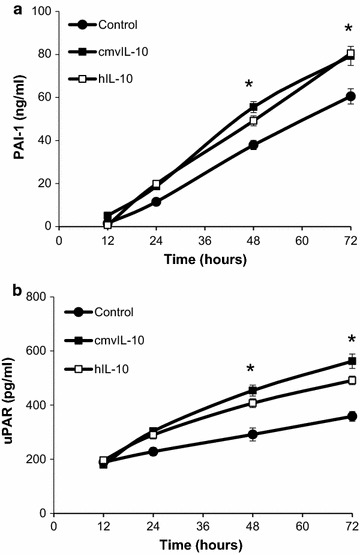
PAI-1 and uPAR levels are elevated upon exposure to cmvIL-10 or hIL-10. **a** MDA-MB-231 cells were cultivated in the presence of 10 ng/ml purified recombinant cmvIL-10 or hIL-10. At the indicated time points, culture supernatants were collected and levels of PAI-1 measured by ELISA. **b** MDA cells were cultured as above and levels of uPAR in the supernatant were determined by ELISA. *Error bars* represent standard error among three replicates for each data point. *p < 0.05. These results are representative of three independent experiments

The most significantly upregulated gene by both cmvIL-10 and hIL-10 was *PAI*-*1*, or plasminogen activator inhibitor 1. PAI-1 is a 43 kDa glycoprotein that inhibits the function of urokinase plasminogen activator (uPA), a serine protease that catalyzes the conversion of inactive plasminogen to plasmin and has been implicated in many aspects of tumor progression [62]. The activity of uPA system is regulated by the receptor uPAR and two endogenous inhibitors, PAI-1 and PAI-2 [62]. PAI-1 is constitutively secreted by many cell types and high levels have been found to inhibit cell adhesion and promote migration [63, 64]. In order to confirm that changes in gene expression identified by the qPCR array correlated with protein expression, MDA cells were treated with cmvIL-10 or hIL-10 and PAI-1 levels measured by ELISA. As expected, PAI-1 was produced by untreated cells, however, the amount of protein secreted was significantly increased by cmvIL-10 after 12 h of exposure (Fig. 3a). After 24 h, both cmvIL-10 and hIL-10 stimulated a significant increase in PAI-1 production, and this was maintained over 72 h. In addition, we examined uPAR protein levels and found that they were also elevated upon exposure to cmvIL-10 or hIL-10 (Fig. 3b). These results demonstrate that expression of two elements of the uPA serine protease system, its receptor uPAR and its serpin inhibitor PAI-1, are significantly increased by cmvIL-10 and hIL-10 in human breast cancer cells.

**Fig. 4:**
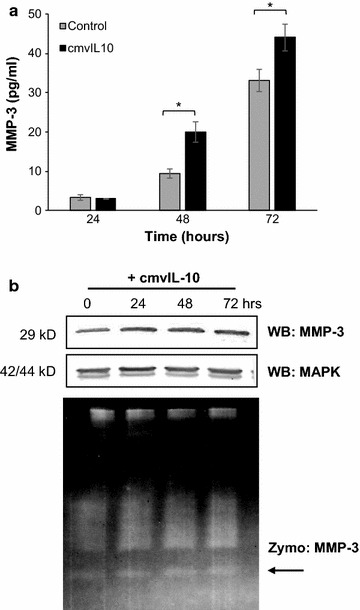
MMP-3 expression and activity are increased by cmvIL-10. **a** MDA-MB-231 cells were cultured in the presence or absence of 10 ng/ml cmvIL-10 for the indicated times and then cell lysates were analyzed by ELISA. *Error bars* SEM, *p < 0.05. **b** MDA cells cultured with 100 ng/ml cmvIL-10 for the indicated times were harvested and lysates examined by western blotting with anti-MMP3 or anti-MAPK as a protein loading control. Lysates from cells receiving the same treatment were analyzed under non-reducing conditions on a 4–16% Zymogram gel (*lower panel*). *Arrow* indicates active MMP-3. These results are representative of three independent experiments

Next we examined *MMP*-*3*, a member of the matrix metalloproteinase family that has the ability degrade many components of the extracellular matrix, such as collagen III-V, and IX-XI, as well as laminins, elastins, fibronectin, vitronectins and proteoglycans [65]. Mouse epithelial mammary cells cultured with MMP-3 had decreased expression of cytokeratin markers and increased expression of vimentin, a clear sign of the epithelial-to-mesenchymal transition (EMT), in which epithelial cells morph into a mesenchymal-type cell to eliminate their connection to the basement membrane and initiate migration towards subsequent intravasation into blood vessels [66]. MMP-3 can also activate other MMPs, and high levels of MMP-3 correlate with poor prognosis in breast cancer patients [67]. MDA cells were treated with cmvIL-10 and then total MMP-3 levels were measured by ELISA. We were unable to detect any MMP-3 in cell supernatants, but cell-associated MMP-3 was detected by analysis of cell lysates. Relatively low levels of MMP-3 were produced by untreated MDA cells, but this amount increased significantly after 48 h of treatment with cmvIL-10 (Fig. 4a). Since MMPs are generally secreted as inactive pro-enzymes that require cleavage to become activated, we further examined MMP-3 by western blotting and zymography. Consistent with the ELISA results, an increase in total MMP-3 protein was observed over time with exposure to cmvIL-10 (Fig. 4b). The amount of active MMP-3 enzyme was also increased by cmvIL-10 treatment, as evidence by increased digestion of casein in the zymogen gel. Taken together, these results indicate that cmvIL-10 promotes increased expression and activation of MMP-3 by breast cancer cells, which is likely to contribute to increased degradation of the ECM and greater risk of metastasis.

**Fig. 5:**
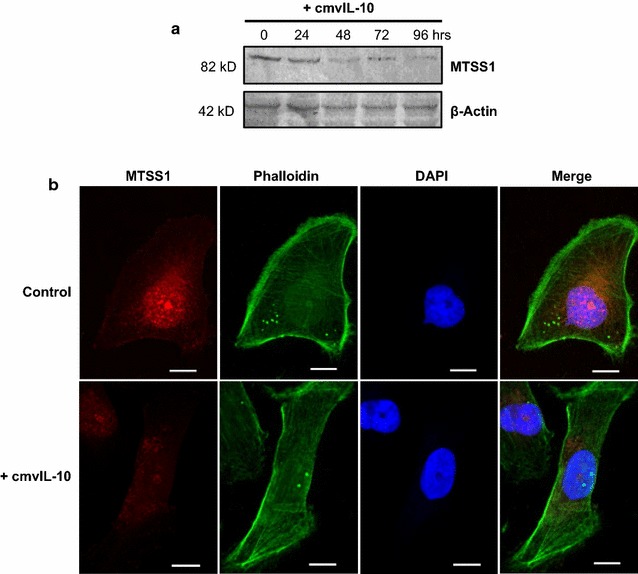
MTSS1 expression is significantly decreased upon exposure to cmvIL-10. **a** MDA-MB-231 cells were cultured with 100 ng/ml cmvIL-10 for the indicated times and lysates examined by western blotting with anti-MTSS1 or anti-β-actin as a protein loading control. **b** MDA cells were seeded onto glass coverslips in the presence of absence of 100 ng/ml cmvIL-10 for 96 h, then fixed, permeabilized, and stained as indicated. *Scale bar* 10 μm. These results are representative of three independent experiments

*MTSS1* was notable as the gene most strongly downregulated by cmvIL-10 treatment. Also known as missing-in-metastasis (MIM), MTSS1 was originally identified as a tumor suppressor gene whose expression was lost in metastatic bladder and prostate cancers [68]. The tumor suppressor works as a scaffold to inhibit the dissociation of cell junctions and to increase adherens junction formation, so when MTSS1 is lost, recruitment of F-actin to the cytoskeleton is reduced, enabling tumor cells to detach from the basement membrane and from neighboring cells. MTSS1 has been found to be inversely correlated to the aggressive invasive potential in several breast cell lines and with overall survival in breast cancer patients [69]. To confirm that the reduced gene expression observed on the PCR array correlated with a decrease in MTSS1 protein levels, immunoblotting was performed on lysates from MDA-MB-231 cells treated with 10 ng/ml cmvIL-10. The expected 82 kD band was detected for MTSS1 in untreated cells and was still visible after 24 h of incubation with cmvIL-10 (Fig. 5a). However, as time progressed, the cmvIL-10-treated samples showed a significant decrease in MTSS1 expression. In contrast, the β-actin bands that serve as a loading control remained constant. We further examined MTSS1 expression via immunofluorescence microscopy and found the protein to be widely distributed throughout the cytoplasm in untreated MDA cells (Fig. 5b), which is consistent with its role in regulating cytoskeletal rearrangement. After exposure to cmvIL-10, dramatic reduction in the amount of MTSS1 protein was observed. This reduction in MTSS1 corresponded to a noticeable change in cellular architecture, as cmvIL-10-treated cells appeared to be thinner and have fewer substrate attachment points. These results demonstrate that treatment with cmvIL-10 reduced the expression of MTSS1 in MDA cells, which could contribute to the increased migration and invasion observed in the presence of cmvIL-10.

## Conclusions

**Fig. 6:**
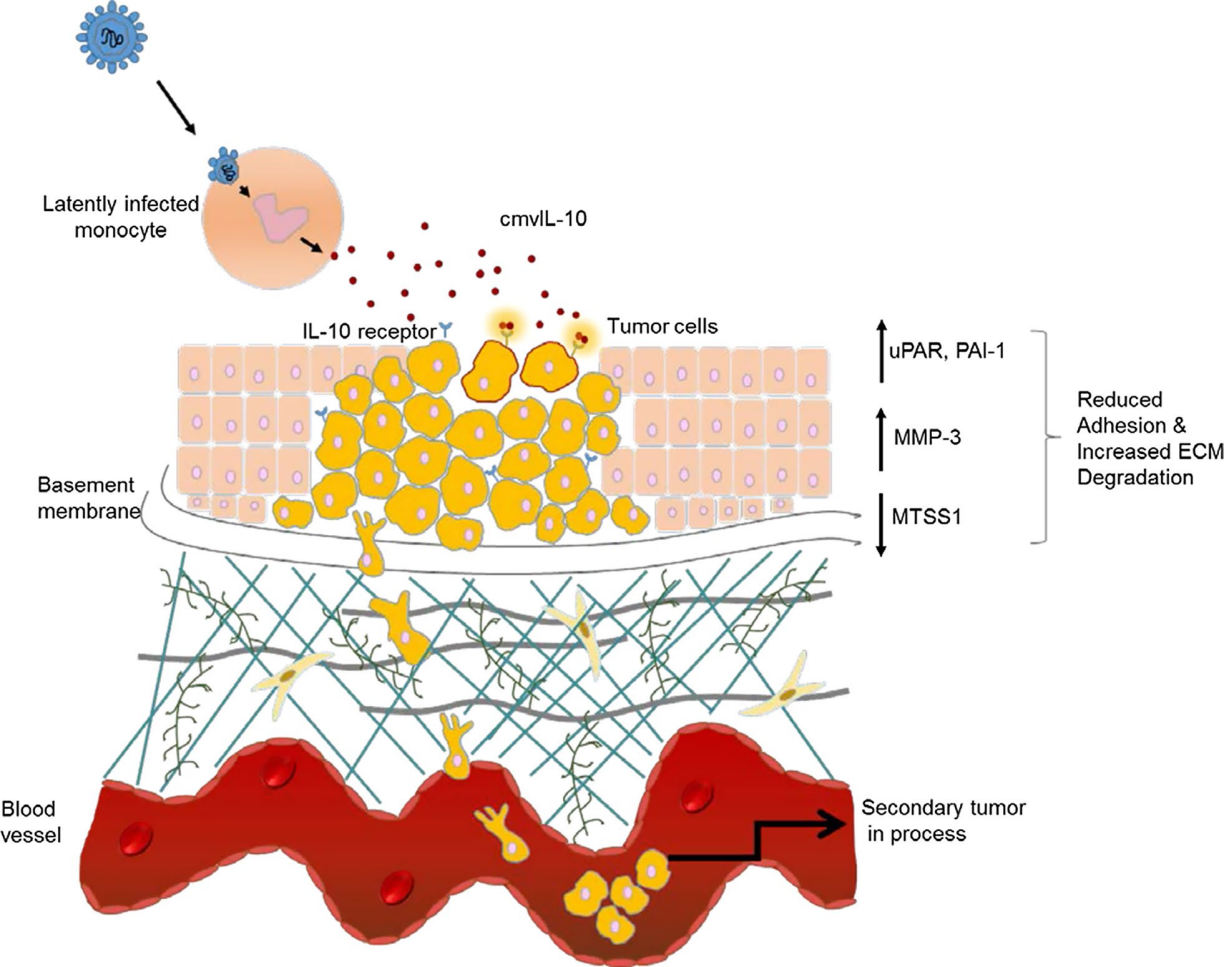
Model depicting possible role of cmvIL-10 in promoting tumor metastasis. A monocyte that is latently infected with HCMV infiltrates a localized tumor, releasing cmvIL-10 that acts on tumor cells expressing the IL-10 receptor. This leads to changes in levels of MTSS1, uPAR and PAI-1, which reduce cell adhesion. Increased levels of uPAR and PAI-1 are strongly associated with increased migration and can also help activate MMP-3. Active MMP-3 degrades proteins in the extracellular matrix, facilitating access for tumor cells to invade surrounding stromal tissue and enter the bloodstream

The results that we present here characterize a new role for cmvIL-10 beyond its well-known function as an immune modulator during HCMV infection [42]. The viral cytokine conferred MDA-MB-231 cells with heightened migration and invasion abilities and is likely to promote the development of breast cancer metastasis. While our studies have been conducted in vitro using cell lines, we propose an in vivo scenario in which latently infected monocyte/macrophages infiltrate the developing tumor and release cmvIL-10 (Fig. 6). Our observations that cmvIL-10 reduces expression of MTSS1, while increasing expression of uPAR, PAI-1, and MMP-3 suggest that the secreted viral cytokine can act directly on breast epithelial cells expressing IL-10R to promote reduced cell–cell adhesion and increased movement, ultimately leading to invasion into the surrounding stromal tissue and entry into bloodstream.

The evidence linking HCMV to breast cancer continues to grow, yet there is still considerable controversy in field. While some groups have found traces of viral DNA or proteins in tumor samples [6, 10, 11], other labs have not been able to detect evidence of HCMV [70, 71]. This may be due to differences in detection technique, sample preparation, and even tumor type. El-Shinawi et al. have found evidence for HCMV in inflammatory breast cancer (IBC), a highly metastatic and aggressive form of breast cancer that is often associated with pregnancy and occurs in at higher frequency in women of Northern African and Egyptian descent [72]. They reported detection of more HCMV DNA in IBC tissues compared to IDC, and higher HCMV IgG titers in IBC patients compared to IDC patients [72]. In a follow-up study, they assessed viral genotypes and found a correlation between mixed phenotypes and disease progression, notably lymphovascular invasion and formation of lymphatic emboli in IBC patients, but not in women with other forms of breast cancer [73]. The findings suggest that HCMV may be more closely associated with specific subtypes of breast cancer.

HCMV can infect a wide range of cell types that may be present within the tumor microenvironment, such as monocyte/macrophages, fibroblasts, epithelial and endothelial cells. While several studies have found evidence for direct infection of tumor cells via IHC staining [10, 11], detection of viral DNA by PCR from fixed tissue samples precludes direct identification of the cell type harboring the virus. It may be that the cell type infected varies from case to case, just like the combination of specific mutations that lead to tumor initiation in a given individual. We favor the notion that immune cells harboring latent HCMV infiltrate the tumor, because monocytes are a well-documented reservoir for HCMV [74–76] and this scenario could lead to significant variability from tumor to tumor depending on the number of infected infiltrating cells and whether they reactivate virus that goes on to infect other cells in the tumor microenvironment. Transcriptional analysis of HCMV-infected monocytes revealed a unique M1/M2 polarization signature that included induction of both M1 type inflammatory cytokines like IL-1, IL-6, and TNFα, as well as upregulation of M2 type cytokines like IL-10 and IL-18 [77]. The presence of these conflicting signals in the tumor environment has been associated with neoplastic progression, suggesting that HCMV could tip the balance in favor of this process [78].

Analysis of the secretome, or proteins produced by HCMV-infected cells, has revealed high levels of both MMP-3 and PAI-1 [79], which is consistent with our observations that cmvIL-10 induces higher expression of both of these proteins. The secretome was found to promote angiogenesis and wound healing, and contained many growth factors, cytokines, chemokines, and enzymes associated with metastasis, including MMP-1, MMP-2, MMP-9, and MMP-10 [79]. Somiari and colleagues were able to detect elevated levels and activity of MMP-2 and MMP-9 in plasma from breast cancer patients, as well as in women determined to be high risk based on Gail Model predictions [80]. This suggests that it may be possible to develop a plasma protein profile with a characteristic signature that identifies individuals likely to develop breast cancer.

Although cmvIL-10 has not yet been quantified in patient serum, measurement of cmvIL-10 and hIL-10 may also have prognostic value in breast cancer. Elevated levels of hIL-10 (27-2134 pg/ml) have already been detected in the serum of some cancer patients and correlate with poor prognosis [81–86], suggesting that hIL-10 may contribute to immune suppression and tumor progression. In vitro, hIL-10 has been found to promote resistance to apoptosis in human breast and lung cancer cell lines [87, 88]. Importantly, recent evidence suggests that cmvIL-10 induces increased expression of hIL-10, potentially amplifying the immune suppressive environment and enabling the invasive spread of tumor cells [60]. Our results show that cmvIL-10 increased the migration and invasive ability of MDA-MB-231 breast cancer cells and affected expression of several metastasis-related genes. Taken together, these findings suggest a new mechanism for HCMV oncomodulation, as secretion of cmvIL-10 is expected to manipulate the tumor microenvironment, enhancing the potential of a developing breast tumor to invade surrounding tissue, and ultimately establish metastatic tumors. Ultimately, it may be that a signature profile of factors like cmvIL-10, hIL-10, MMPs, and PAI-1 could have predictive or prognostic value for breast cancer. If HCMV is truly involved in promoting tumor progression, chemotherapy treatment regimens that include anti-cmvIL-10 specific antibodies or even anti-viral drugs may help improve the overall survival of cancer patients.

## Additional file

**Additional file 1.** Transcriptional profiling of MDA-MB-231 cells exposed to cmvIL-10 or hIL-10.

## Abbreviations

cDNA: complementary deoxyribonucleic acid
COX-2: cyclooxygenase-2
DAPI: 4′,6-diamidino-2-phenylindole
DCIS: ductal carcinoma in situ
ECM: extracellular matrix
EBV: Epstein–Barr virus
EGF: epidermal growth factor
ELISA: enzyme-linked immunosorbent assay
FBS: fetal bovine serum
GBM: glioblastoma multiforme
HCMV: human cytomegalovirus
HFF: human foreskin fibroblast
HHV-8: human herpesvirus 8
IBC: inflammatory breast cancer
IDC: infiltrating ductal carcinoma
IE: immediate early
IgG: immunoglobulin G
IHC: immunohistochemistry
MHC: major histocompatibility complex
MMP: matrix metalloproteinase
MOI: multiplicity of infection
MTSS1: metastasis suppressor 1
NAb: neutralizing antibody
PAI-1: plasminogen activator inhibitor 1
PCR: polymerase chain reaction
RNA: ribonucleic acid
TRITC: tetramethylrhodamine
uPAR: urokinase plasminogen receptor
VEGF: vascular endothelial growth factor (VEGF)
