# Supplementary material for: Human cytomegalovirus interleukin-10 enhances matrigel invasion of MDA-MB-231 breast cancer cells
Source: Cancer Cell Int. 2017 Feb 13;17:24. doi: 10.1186/s12935-017-0399-5 (PMC5307693; doi:10.1186/s12935-017-0399-5)
Supplement: Supplementary file 1 — Additional file 1. Transcriptional profiling of MDA-MB-231 cells exposed to cmvIL-10 or hIL-10. [file 12935_2017_399_MOESM1_ESM.docx]

**Supplemental Table 1: Transcriptional Profiling of MDA-MB-231 Cells Exposed to cmvIL-10 or hIL-10**

**Fold Change vs. Mock**

| **Unigene** | **Refseq** | **Symbol** | **Description** | **cmvIL-10** | **hIL-10** |
| --- | --- | --- | --- | --- | --- |
| Hs.158932 | NM_000038 | APC | Adenomatous polyposis coli | 0.9681 | 1.0052 |
| Hs.100426 | NM_015399 | BRMS1 | Breast cancer metastasis suppressor 1 | 0.8029 | 1.1154 |
| Hs.251526 | NM_006273 | CCL7 | Chemokine (C-C motif) ligand 7 | 0.8113 | 0.9314 |
| Hs.502328 | NM_000610 | CD44 | CD44 molecule (Indian blood group) | 1.1473 | 1.3402 |
| Hs.527778 | NM_002231 | CD82 | CD82 molecule | 0.6917 | 1.1019 |
| Hs.461086 | NM_004360 | CDH1 | Cadherin 1, type 1, E-cadherin (epithelial) | 0.6288 | 0.7618 |
| Hs.116471 | NM_001797 | CDH11 | Cadherin 11, type 2, OB-cadherin (osteoblast) | 0.9033 | 1.0425 |
| Hs.171054 | NM_004932 | CDH6 | Cadherin 6, type 2, K-cadherin (fetal kidney) | 0.8113 | 0.9314 |
| Hs.512599 | NM_000077 | CDKN2A | Cyclin-dependent kinase inhibitor 2A (melanoma, p16, inhibits CDK4) | 0.8113 | 0.9314 |
| Hs.162233 | NM_001273 | CHD4 | Chromodomain helicase DNA binding protein 4 | 0.8970 | 1.1115 |
| Hs.508716 | NM_001846 | COL4A2 | Collagen, type IV, alpha 2 | 1.0929 | 1.5131 |
| Hs.143212 | NM_003650 | CST7 | Cystatin F (leukocystatin) | 0.7149 | 0.8858 |
| Hs.208597 | NM_001328 | CTBP1 | C-terminal binding protein 1 | 0.6245 | 0.9576 |
| Hs.534797 | NM_001903 | CTNNA1 | Catenin (cadherin-associated protein), alpha 1, 102kDa | 0.8816 | 0.9428 |
| Hs.632466 | NM_000396 | CTSK | Cathepsin K | 0.8043 | 0.8482 |
| Hs.716407 | NM_001912 | CTSL1 | Cathepsin L1 | 0.8212 | 0.8981 |
| Hs.522891 | NM_000609 | CXCL12 | Chemokine (C-X-C motif) ligand 12 | 0.8113 | 0.9314 |
| Hs.846 | NM_001557 | CXCR2 | Chemokine (C-X-C motif) receptor 2 | 0.3804 | 0.5885 |
| Hs.593413 | NM_003467 | CXCR4 | Chemokine (C-X-C motif) receptor 4 | 0.8084 | 0.8751 |
| Hs.22393 | NM_003677 | DENR | Density-regulated protein | 0.9816 | 0.9626 |
| Hs.523329 | NM_004442 | EPHB2 | EPH receptor B2 | 0.6693 | 0.9028 |
| Hs.434059 | NM_001986 | ETV4 | Ets variant 4 | 0.5418 | 1.0175 |
| Hs.374477 | NM_005243 | EWSR1 | Ewing sarcoma breakpoint region 1 | 0.9465 | 1.3104 |
| Hs.481371 | NM_005245 | FAT1 | FAT tumor suppressor homolog 1 (Drosophila) | 1.2752 | 1.4414 |
| Hs.165950 | NM_002011 | FGFR4 | Fibroblast growth factor receptor 4 | 0.5362 | 0.9693 |
| Hs.646917 | NM_002020 | FLT4 | Fms-related tyrosine kinase 4 | 0.8113 | 1.0317 |
| Hs.203717 | NM_002026 | FN1 | Fibronectin 1 | 1.1044 | 1.2100 |
| Hs.333418 | NM_014164 | FXYD5 | FXYD domain containing ion transport regulator 5 | 0.9400 | 1.0479 |
| Hs.82963 | NM_000825 | GNRH1 | Gonadotropin-releasing hormone 1 (luteinizing-releasing hormone) | 0.9970 | 1.1647 |
| Hs.396530 | NM_000601 | HGF | Hepatocyte growth factor (hepapoietin A; scatter factor) | 0.8113 | 0.9314 |
| Hs.44227 | NM_006665 | HPSE | Heparanase | 0.8620 | 0.8645 |
| Hs.37003 | NM_005343 | HRAS | V-Ha-ras Harvey rat sarcoma viral oncogene homolog | 1.0075 | 1.0625 |
| Hs.90753 | NM_006410 | HTATIP2 | HIV-1 Tat interactive protein 2, 30kDa | 0.7439 | 0.9138 |
| Hs.160562 | NM_000618 | IGF1 | Insulin-like growth factor 1 (somatomedin C) | 0.8635 | 0.9794 |
| Hs.83077 | NM_001562 | IL18 | Interleukin 18 (interferon-gamma-inducing factor) | 0.7362 | 0.7526 |
| Hs.126256 | NM_000576 | IL1B | Interleukin 1, beta | 0.6266 | 0.6771 |
| Hs.524484 | NM_002206 | ITGA7 | Integrin, alpha 7 | 0.5609 | 0.8796 |
| Hs.218040 | NM_000212 | ITGB3 | Integrin, beta 3 (platelet glycoprotein IIIa, antigen CD61) | 1.0198 | 1.0317 |
| Hs.95008 | NM_002256 | KISS1 | KiSS-1 metastasis-suppressor | 1.1394 | 1.5856 |
| Hs.208229 | NM_032551 | KISS1R | KISS1 receptor | 0.8113 | 0.9314 |
| Hs.505033 | NM_004985 | KRAS | V-Ki-ras2 Kirsten rat sarcoma viral oncogene homolog | 1.0304 | 0.9931 |
| Hs.599039 | NM_006500 | MCAM | Melanoma cell adhesion molecule | 0.7688 | 1.0353 |
| Hs.484551 | NM_002392 | MDM2 | Mdm2 p53 binding protein homolog (mouse) | 0.9531 | 0.9461 |
| Hs.132966 | NM_000245 | MET | Met proto-oncogene (hepatocyte growth factor receptor) | 0.9833 | 1.1173 |
| Hs.444986 | NM_006838 | METAP2 | Methionyl aminopeptidase 2 | 1.1295 | 1.0736 |
| Hs.651869 | NM_002410 | MGAT5 | Mannosyl (alpha-1,6-)-glycoprotein beta-1,6-N-acetyl-glucosaminyltransferase | 1.1795 | 1.3590 |
| Hs.2258 | NM_002425 | MMP10 | Matrix metallopeptidase 10 (stromelysin 2) | 0.5980 | 0.6866 |
| Hs.143751 | NM_005940 | MMP11 | Matrix metallopeptidase 11 (stromelysin 3) | 0.7026 | 1.0867 |
| Hs.2936 | NM_002427 | MMP13 | Matrix metallopeptidase 13 (collagenase 3) | 0.8127 | 0.9777 |
| Hs.513617 | NM_004530 | MMP2 | Matrix metallopeptidase 2 (gelatinase A) | -1.1101 | 1.5157 |
| Hs.375129 | NM_002422 | MMP3 | Matrix metallopeptidase 3 (stromelysin 1, progelatinase) | 2.7478 | 0.8080 |
| Hs.2256 | NM_002423 | MMP7 | Matrix metallopeptidase 7 (matrilysin, uterine) | 0.9416 | 0.8123 |
| Hs.297413 | NM_004994 | MMP9 | Matrix metallopeptidase 9 (gelatinase B) | 1.0233 | 0.9347 |
| Hs.525629 | NM_004689 | MTA1 | Metastasis associated 1 | 0.7388 | 0.9395 |
| Hs.700429 | NM_014751 | MTSS1 | Metastasis suppressor 1 | -3.2818 | 1.2397 |
| Hs.202453 | NM_002467 | MYC | V-myc myelocytomatosis viral oncogene homolog (avian) | 0.8113 | 0.7423 |
| Hs.437922 | NM_005376 | MYCL1 | V-myc myelocytomatosis viral oncogene homolog 1, lung carcinoma derived (avian) | 0.5260 | 1.0644 |
| Hs.187898 | NM_000268 | NF2 | Neurofibromin 2 (merlin) | 1.0742 | 1.3496 |
| Hs.118638 | NM_000269 | NME1 | Non-metastatic cells 1, protein (NM23A) expressed in | 1.0358 | 1.1000 |
| Hs.9235 | NM_005009 | NME4 | Non-metastatic cells 4, protein expressed in | 0.8226 | 0.9965 |
| Hs.279522 | NM_006981 | NR4A3 | Nuclear receptor subfamily 4, group A, member 3 | 0.5389 | 0.8151 |
| Hs.466871 | NM_002659 | PLAUR | Plasminogen activator, urokinase receptor | 1.5891 | 1.8628 |
| Hs.409965 | NM_002687 | PNN | Pinin, desmosome associated protein | 1.3088 | 1.2901 |
| Hs.500466 | NM_000314 | PTEN | Phosphatase and tensin homolog | 0.7809 | 0.8645 |
| Hs.408528 | NM_000321 | RB1 | Retinoblastoma 1 | 0.9548 | 0.9298 |
| Hs.494178 | NM_006914 | RORB | RAR-related orphan receptor B | 0.6953 | 0.8827 |
| Hs.449909 | NM_002295 | RPSA | Ribosomal protein SA | 0.9631 | 1.0886 |
| Hs.414795 | NM_000602 | SERPINE1 | Serpin peptidase inhibitor, plasminogen activator inhibitor type 1 | 2.6818 | 3.0262 |
| Hs.436687 | NM_003011 | SET | SET nuclear oncogene | 0.9664 | 0.9234 |
| Hs.12253 | NM_005901 | SMAD2 | SMAD family member 2 | 0.6869 | 0.7859 |
| Hs.75862 | NM_005359 | SMAD4 | SMAD family member 4 | 0.8384 | 0.8279 |
| Hs.195659 | NM_005417 | SRC | V-src sarcoma (Schmidt-Ruppin A-2) viral oncogene homolog (avian) | 0.5297 | 0.9760 |
| Hs.514451 | NM_001050 | SSTR2 | Somatostatin receptor 2 | 0.5556 | 0.7120 |
| Hs.371720 | NM_003177 | SYK | Spleen tyrosine kinase | 0.8590 | 0.9314 |
| Hs.475018 | NM_005650 | TCF20 | Transcription factor 20 (AR1) | 0.7823 | 0.9676 |
| Hs.645227 | NM_000660 | TGFB1 | Transforming growth factor, beta 1 | 0.7173 | 0.9931 |
| Hs.633514 | NM_003255 | TIMP2 | TIMP metallopeptidase inhibitor 2 | 0.7596 | 0.8601 |
| Hs.644633 | NM_000362 | TIMP3 | TIMP metallopeptidase inhibitor 3 | 0.8155 | 0.7631 |
| Hs.591665 | NM_003256 | TIMP4 | TIMP metallopeptidase inhibitor 4 | 0.7324 | 0.9122 |
| Hs.478275 | NM_003810 | TNFSF10 | Tumor necrosis factor (ligand) superfamily, member 10 | 0.8169 | 0.7955 |
| Hs.654481 | NM_000546 | TP53 | Tumor protein p53 | 0.6180 | 0.8873 |
| Hs.155942 | NM_002420 | TRPM1 | Transient receptor potential cation channel, subfamily M, member 1 | 0.8113 | 0.9314 |
| Hs.160411 | NM_000369 | TSHR | Thyroid stimulating hormone receptor | 0.8113 | 0.9314 |
| Hs.73793 | NM_003376 | VEGFA | Vascular endothelial growth factor A | 0.5541 | 0.5987 |
| Hs.520640 | NM_001101 | ACTB | Actin, beta | 1.0986 | 1.3287 |
| Hs.534255 | NM_004048 | B2M | Beta-2-microglobulin | 0.9597 | 0.9897 |
| Hs.592355 | NM_002046 | GAPDH | Glyceraldehyde-3-phosphate dehydrogenase | 0.9335 | 1.2121 |
| Hs.412707 | NM_000194 | HPRT1 | Hypoxanthine phosphoribosyltransferase 1 | 1.2796 | 1.3827 |
| Hs.546285 | NM_001002 | RPLP0 | Ribosomal protein, large, P0 | 1.0376 | 1.0000 |
